# Physical Exercise and the Renin Angiotensin System: Prospects in the COVID-19

**DOI:** 10.3389/fphys.2020.561403

**Published:** 2020-10-15

**Authors:** Fabiana S. Evangelista

**Affiliations:** School of Arts, Science and Humanities, University of São Paulo, São Paulo, Brazil

**Keywords:** Coronavirus, molecular mechanism, angiotensin converting enzyme 2, exercise training, treatment, cardiometabolic diseases

## Abstract

Recent reports have shown that the renin angiotensin system (RAS) plays an important role in the Coronavirus disease 2019 (COVID-19) because the angiotensin converting enzyme 2 is the receptor for the severe acute respiratory syndrome coronavirus 2. In addition, the balance of RAS components can be involved in the pathogenesis and progression of COVID-19, especially in patients with metabolic and cardiovascular diseases. On the other hand, physical exercise is effective to prevent and to counteract the consequences of such diseases and one of the biological mediators of the exercise adaptation is the RAS. This review was designed to highlight the connection between COVID-19 and RAS, and to discuss the role of the RAS as a mediator of the benefits of physical exercise in COVID-19 pandemic.

## Introduction

Early reports suggested that patients with severe Coronavirus disease 2019 (COVID-19) were more likely to have a history of hypertension, chronic kidney disease, cardiovascular disease (CVD), or diabetes mellitus (DM) than those with milder disease (Lai et al., 2020; Zhou et al., 2020). Due to the severe acute respiratory distress syndrome with high morbidity and mortality, the search for uncover the mechanisms involved in the pathogenesis and progression of COVID-19 has become urgent. Some evidence suggest a role of the renin angiotensin system (RAS), since an enzyme of the RAS named angiotensin converting enzyme 2 (ACE2) is the receptor for the severe acute respiratory syndrome coronavirus 2 (SARS-CoV-2) protein in the alveolar epithelial cells in the lungs (Li et al., 2003; Hoffmann et al., 2020).

Because of the virus infection depend on ACE2, pharmacological manipulation of the RAS through angiotensin converting enzyme (ACE) inhibitors, angiotensin receptor blockers (ARBs), and soluble recombinant ACE2 proteins administration have been discussed as a potential therapy for COVID-19 (Batlle et al., 2020; South et al., 2020). While the preventive or therapeutic medical interventions for COVID-19 infection are still the big challenges of science, the recommendation to avoid social interactions is crucial as well as to maintain an adequate health status. Thus, the practice of physical exercise (PE) during the quarantine is a critical public health topic in facing the pandemic (Chen et al., 2020; Jiménez-Pavón et al., 2020) since the effectiveness of PE to prevent and to counteract the consequences of cardiovascular and metabolic diseases is widely recognized (Cornelissen and Smart, 2013; Madden, 2013). In addition, reducing sedentary behavior in times of social isolation through home-based exercise programs seems to be feasible, safe, and effective, and tools such as physical activity trackers and applications for smartwatches and phones can be used to deliver the supervised PE (Peçanha et al., 2020; Schwendinger and Pocecco, 2020). Remotely supervised home-based exercise can be a strategy to reduce health deficits arising from coping with the pandemic also in healthy individuals (Schwendinger and Pocecco, 2020) or with CVDs (Safiyari-Hafizi et al., 2016; Avila et al., 2018).

Evidence in the literature showed that biological mediators, such as the RAS pathway, are involved in the exercise adaptation (Frantz et al., 2018; Echeverría-Rodríguez et al., 2020). Considering that the RAS can be carefully manipulated to mitigate the severe acute respiratory syndrome induced tissue injuries, which represents a potential target for therapeutic intervention (Kuba et al., 2005), and that part of the effects of PE can be mediated by the RAS, the purpose of this review is: (1) to highlight the connection between COVID-19 and RAS and (2) to discuss the role of the RAS as a mediator of the benefits of PE in COVID-19 pandemic.

## The Connection Between Covid-19 and the RAS

**Figure 1:**
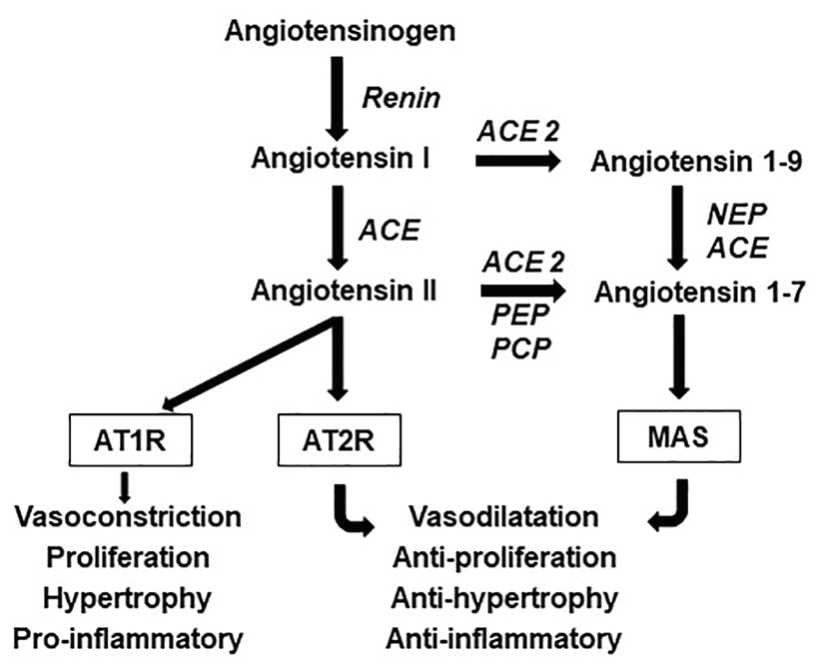
Components and effects of the renin angiotensin system (RAS). The angiotensinogen is cleaved by renin to form angiotensin I. The angiotensin-converting enzyme (ACE) cleaves angiotensin I to form the angiotensin II (Ang II), which can be catabolized by angiotensin converting enzyme 2 (ACE2) into angiotensin 1–7 (Ang 1–7), another active peptide of this system which typically opposes the actions of Ang II. Most effects of Ang II are mediated by the angiotensin type 1 receptor (AT1R), however, Ang II can also bind to the angiotensin type 2 receptor (AT2R), which generally exhibits opposing effects to those at the AT1R. Ang 1–7 acts *via* the Mas receptor. Endopeptidases and carboxypeptidase also cleave Ang II and Ang 1–9 to form Ang 1–7. NEP, neutral-endopeptidase; PEP, prolyl-endopeptidase; and PCP, prolyl-carboxypeptidase.

The RAS corresponds to a complex endocrine, paracrine, and autocrine system which plays an important function, controlling cardiovascular function and metabolic homeostasis (Figure 1). One of axis of the RAS includes ACE, angiotensin II (Ang II) and AT1 receptor (ACE/Ang II/AT1R axis), which is associated to vasoconstriction, cell proliferation, organ hypertrophy, and sodium retention. Most effects of Ang II are mediated by AT1R, however, Ang II can also bind to the angiotensin type 2 receptor (AT2R), which generally exhibits opposing effects to those at AT1R (Santos et al., 2008).

The hyperactivated status of ACE/Ang II/AT1R axis is observed in metabolic diseases such as obesity, DM, and inflammation (Tabony et al., 2011; Slamkova et al., 2016) and in CVD (Putnam et al., 2012). This axis is also associated with the development of pulmonary hypertension and fibrosis (Ferreira et al., 2009; Shenoy et al., 2010). Therefore, ACE inhibitors and ARBs are usually prescribed to patients worldwide to treat these diseases (Putnam et al., 2012).

A counter-regulatory RAS axis consists of ACE2, an ACE homologue enzyme, angiotensin 1–7 (Ang 1–7), and the Mas receptor (ACE2/Ang 1–7/Mas; Figure 1). This axis, when activated, induces anti-inflammatory, vasodilator, antiproliferative, cardioprotective, and renoprotective responses (Santos et al., 2018). In an interstitial lung disease animal model, the increase of this axis can reduce the excessive deposition of pulmonary collagen, the systolic pressure of the right ventricle, the right ventricular fibrosis, and pulmonary vascular remodeling (Ferreira et al., 2009; Silva et al., 2013). Also, it reduces body weight and improves lipid profile, increases glucose uptake, and reduces oxidative stress (Santos et al., 2010; Ramalingam et al., 2017). Because of these responses, the ACE2/Ang 1–7/Mas axis counteracts the deleterious effect of ACE/Ang II/AT1R axis and has been investigated as a target for reducing metabolic diseases and CVD (Rabelo et al., 2011; Ramalingam et al., 2017).

There are two types of ACE2: the soluble and the full-length. The soluble type of ACE2 lacks the membrane anchor and circulates in small amounts in the blood (Wysocki et al., 2010). The full-length type, which is expressed in different organs including the lungs, mediates the connection between COVID-19 and the RAS because it contains a structural transmembrane domain that acts as the receptor for the SARS-CoV-2 spike protein, which facilitates the entry of the virus into host cells, viral replication, and cell-to-cell transmission (Li et al., 2003; Hoffmann et al., 2020). The binding of the SARS-CoV-2 spike protein to ACE2 is followed by proteolytic cleavage and viral entry, which induces ACE2 internalization and shedding, and reduced expression of ACE2. Thereby, the effect of reducing tissue ACE2 is to decrease Ang 1–7 and increase Ang II levels, resulting in systemic RAS imbalance (South et al., 2020). In this scenario, there may be a predominance of pro-inflammatory function relative to the anti-inflammatory function of RAS, which can explain the role of the RAS in COVID-19 progression (Tseng et al., 2020). Also, the higher mortality with COVID-19 may be associated with elevated plasma Ang II levels observed in patients with chronic inflammatory diseases, such as pulmonary arterial hypertension, diabetes, and obesity, (Price et al., 1999; South et al., 2019; Sandoval et al., 2020).

The manipulation of the RAS has been discussed as a potential therapy for COVID-19. An example is the use of ACE inhibitors and ARBs that are capable of modulating both systemic and tissue RAS. While in a cohort of 12 COVID-19 patients, circulating Ang II levels were markedly elevated compared to healthy controls (linearly correlated with viral load; Liu et al., 2020), it was showed that decreasing Ang II with ACE inhibitors and ARBs can improve Ang 1–7 and attenuate inflammation, fibrosis, and lung injury (South et al., 2020). In addition, the use of ARBs by experimental models of acute lung injury, including a model of SARS-CoV infection, may alleviate the disease by increasing ACE2 expression and attenuating Ang II-mediated acute lung injury (Kuba et al., 2005). Despite the evidence, the RAS inhibition in COVID-19 patients with CVD and the use of ARBs by patients already infected by SARS-CoV-2 need further investigation.

Another example considers the ACE2 as a target in preventing and treating chronic inflammation and inflammatory diseases, as highlighted by COVID-19 pandemic. Batlle et al. (2020) hypothesized that soluble recombinant ACE2 proteins administration could attenuate coronavirus infection by prevent binding of the viral particle to the structural transmembrane domain of full-length ACE2 in a competitive way. In addition, as discussed by Gheblawi et al. (2020), maintaining ACE2 levels in patients with or predisposed to risk factors for CVD such as DM, hypertension, and obesity wards off the advancement of these comorbidities in instances where the patient contracts SARS-CoV-2 by maintaining a level of ACE2/Ang1–7/Mas negative counter-regulation.

## Physical Exercise and the RAS

Regular PE is as important as staying at home during the COVID-19 crisis. It is a non-pharmacological tool that promotes beneficial effects on blood glucose level, arterial blood pressure, lipid profile, and body composition. PE also improves the balance of pro and anti-inflammatory cytokines and the immune system response. Thus, PE has been widely recommended to prevent and to treat DM, dyslipidemia, obesity, hypertension, and CVD (Cornelissen and Smart, 2013; Madden, 2013; Higa et al., 2014). Thus, it has been recommended to maintain the routine daily exercise and to avoid sedentary behaviors. But the question is: what are the mechanisms underlying health-related effects of PE? There are certainly several mechanisms and what will be discussed here is the role of the RAS.

**Figure 2:**
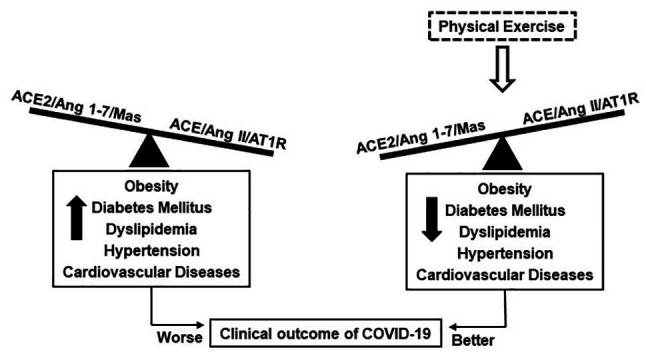
Possible effect of physical exercise in Coronavirus disease 2019 (COVID-19) can be associated with changes in the balance of RAS axes. The unbalance of the RAS is observed in individuals with cardiometabolic diseases, which are more susceptible to severe COVID-19. Physical exercise improves the balance of the RAS toward the protective arm ACE2/Ang 1–7/Mas axis, which is associated with cardiometabolic diseases reduction and possibly better clinical outcome of COVID-19.

The effect of PE on the RAS has already been reviewed in the literature by different researchers (Fiorino and Evangelista, 2014; Goessler et al., 2016; Nunes-Silva et al., 2017; Frantz et al., 2018). The studies have shown that PE induces systemic and tissue ACE/Ang II/AT1R axis downregulation, ACE2/Ang 1–7/Mas axis upregulation, and a shift in the RAS toward the ACE2/Ang (1–7)/Mas axis. These findings suggest that the benefits of PE may be mediated by a shift of the RAS balance toward the protective arm ACE2/Ang 1–7/Mas axis relative to ACE/Ang II/AT1R axis (Figure 2). In this context, the effects of PE on the RAS are in line with sought by the pharmacological strategies of RAS manipulation for the treatment of COVID-19, which can possibly reduce the severity of clinical outcome.

The role of the RAS in mediating the effects of PE on cardiovascular response and to reduce cardiovascular risk is widely known in the literature. In healthy man, aerobic PE suppressed the plasma renin and Ang II and induced substantial gain of aerobic peak oxygen uptake and physical working capacity (Hespel et al., 1988). Healthy animals submitted to aerobic moderate swimming training increased AT1R and AT2R expression, decreased ACE and Ang II levels, and increased ACE2 and Ang (1–7) levels in the heart. These results were associated with physiological left ventricular hypertrophy and resting bradycardia, which are markers of cardiac improvement (Fernandes et al., 2011). Lower ACE and Ang II levels can diminish the vascular resistance and increase cardiac flow, while higher ACE2 and Ang 1–7 can improve the vasodilator response by mediating the release of vasoactive factors, such as NO, prostaglandins, and bradykinin (Li et al., 1997; Carvalho et al., 2007).

Studies have showed the effect of aerobic PE on the RAS in spontaneous hypertensive rats. These animals presented Ang 1–7 and Mas receptor upregulation in the heart and improved cardiac response (Gomes-Filho et al., 2008), increased Mas receptor expression in aorta, thereby improving the vasodilator effect of Ang 1–7 (Silva et al., 2011). They also decreased angiotensinogen expression and AngII/Ang 1–7 ratio in the renal artery, thereby reducing the vasoconstrictor axis and arterial blood pressure (Silva et al., 2015). In addition, Silva et al. (2017) found that aerobic PE decreased left ventricular and plasma Ang II and increased left ventricular and plasma Ang 1–7 levels, normalized oxidative stress, augmented antioxidant defense, and reduced both collagen deposition and inflammatory profile. These changes were accompanied by reduction in arterial blood pressure.

Gomes-Santos et al. (2014) studied the RAS in the skeletal muscle of trained heart failure rats and revealed that aerobic PE normalized ACE2 and reduced ACE in plasma but did not change in the skeletal muscle. Ang 1–7/Ang II ratio increased in the plasma, and Ang 1–7 and Mas receptor were higher in the skeletal muscle. The animals did not improve echocardiographic parameters or peak oxygen uptake. However, they significantly increased tolerance to physical effort and this response is a therapeutic goal for individuals and animals with CVD (Takada et al., 2014; Yokota et al., 2017).

The role of RAS in mediating the effects of PE on glucose metabolism and lipid profile to reduce metabolic diseases is described in the literature. It was shown that one bout of exercise (swimming) increased Ang1–7/Ang II ratio in muscle of healthy rats and improved the insulin sensitivity due to Ang 1–7 acting through Mas receptor (Echeverría-Rodríguez et al., 2020). Furthermore, Ang 1–7 participates in the enhancement of vascular insulin sensitivity after an exercise session (Gallardo-Ortiz et al., 2020). Magalhães et al. (2020) observed that individuals submitted to high-intensity interval exercise increased plasma ACE2 and urinary ACE and Ang 1–7 levels. However, moderate-intensity continuous exercise decreased ACE level in the plasma and elevated urinary concentration of ACE2 and Ang 1–7. The increase of urinary Ang 1–7 was greater in moderate-intensity protocol. Both protocols did not change plasma levels of Ang II and Ang 1–7, however, they reduced capillary blood glucose. The authors concluded that acute PE favored the balance toward the activation of ACE2/Ang 1–7 axis, which was greater in moderate-intensity protocol.

Aerobic PE reduced body mass gain, intra-abdominal fat pad, and leptin levels, improved body composition and inflammatory cytokine, glucose tolerance, and insulin resistance in obese rats (Frantz et al., 2017). The authors also showed reduction in total cholesterol and triacylglycerol levels. The protective effects against obesity, insulin resistance, and DM in trained animals were associated with lower AT1 receptor expression and ACE/ACE2 ratio, higher Mas receptor protein expression, and a shifted RAS balance toward the ACE2/Mas receptor axis in skeletal muscle (Frantz et al., 2017).

In a previous study of our group, aerobic PE prevented body weight gain and adiposity, glucose intolerance, and insulin resistance in mice fed a cafeteria diet. These responses were associated with lower insulin signaling proteins and higher lipolysis signaling proteins in the subcutaneous white adipose tissue. Regarding the circulating RAS, both ACE and ACE2 activities did not change and the Ang 1–7 concentration increased (Américo et al., 2019). The improvement of circulating Ang 1–7 is related to better metabolic response since the chronic systemic Ang 1–7 administration tested by other authors provided significant reduction in body weight and adipose tissue mass, decreased total cholesterol and triglycerides, increased insulin sensitivity, glucose tolerance, and decreased the expression of proinflammatory cytokines mRNA (Bilman et al., 2012; Santos et al., 2012; Loloi et al., 2018). In the subcutaneous white adipose tissue, no differences were found in ACE activity and Ang II content, however, both AT1R and AT2R expression were increased. Also, we observed an increase in the ACE2 activity and Mas receptor expression but no changes in Ang 1–7 level. The partial modulation of the RAS axes allowed to conclude that the prevention of obesity and insulin resistance could not be completely associated with the RAS modulation in the subcutaneous white adipose tissue (Américo et al., 2019). Because of the RAS components expression in multiple tissues, changes in a specific tissue do not necessarily reflect the RAS of whole tissues.

Disturbances in the glucose and lipid metabolism are also observed in other metabolic diseases, such as non-alcoholic fatty liver disease (NAFLD) and hepatic steatosis. Both are associated with obesity, increased cardiovascular risk, chronic kidney disease, insulin resistance, and DM. It has been shown that PE is an important tool to prevent and to treat NAFLD (Stevanovic et al., 2020) and the RAS may be involved in this response. Frantz et al. (2017) found that aerobic PE increased ACE2 protein, Ang 1–7, and Mas receptor in the liver of animals fed a high fructose diet. They also showed normalization of ACE and Ang II in the liver, but the systemic RAS did not change. These results were associated with the prevention of hepatic steatosis, triacylglycerol, and glycogen accumulation in the liver, and reduced pro-inflammatory cytokines levels.

Studies showing the effect of PE in pulmonary RAS are scarce in the literature. Only one showed that aerobic PE associated or not with an ACE2 activator (Diminazene) reduced pulmonary fibrosis in an interstitial lung disease animal model (Prata et al., 2017). The effectiveness of isolated PE as a treatment was evident, however, the conclusion highlighted that PE associated with the activation of ACE2 potentially reduces pulmonary fibrosis. Despite providing important findings, the study did not evaluate the RAS components in the blood and lungs. There are still gaps about the effects of PE on pulmonary RAS and the association with morphological and functional characteristics in physiological and pathological situations.

Given the consistent data demonstrating the effects of PE, it is possible to highlight two ways in which PE can have important implications for COVID-19: first, reducing the metabolic and cardiovascular risk factors, and second, improving the balance of the RAS by increasing the ACE2/Ang (1–7)/Mas axis and reducing the ACE/Ang II/AT1R axis. Further experimental and clinical studies are needed to clarify the precise mechanisms of PE in COVID-19, but these findings strengthen the recommendation for PE during the pandemic period and should be considered a critical public health in facing the pandemic.

## Conclusion

Collectively, the evidence presented here allows to reinforce the importance of PE to improve the health status, and to prevent and to treat diseases that are determinant in the clinical outcome of individuals with COVID-19. Furthermore, the elucidation of the RAS balance improvement as a mechanism induced by PE corroborate the therapeutics targets that are under investigation for COVID-19 and it should be considered by clinicians and epidemiologists. Finally, further investigations still need to be done about the effect of PE on COVID-19, which will certainly contribute to the progress of clinical management.

## Author Contributions

FE contributed to the conceptualization, preparation of the original draft, and final editing.

### Conflict of Interest

The author declares that the research was conducted in the absence of any commercial or financial relationships that could be construed as a potential conflict of interest.
